# Evidence of extensive cellular immune response after SARS-CoV-2 vaccination in ocrelizumab-treated patients with multiple sclerosis

**DOI:** 10.1186/s42466-021-00158-5

**Published:** 2021-11-22

**Authors:** Mosche Pompsch, Neslinur Fisenkci, Peter A. Horn, Markus Kraemer, Monika Lindemann

**Affiliations:** 1 Department of Neurology, Alfried Krupp Hospital, Alfried-Krupp-Straße 21, 45130 Essen, Germany; 2 Institute for Transfusion Medicine, University Hospital Essen, University of Duisburg-Essen, Essen, Germany; 3 Department of Neurology, Medical Faculty of Heinrich-Heine-University, Düsseldorf, Germany

**Keywords:** Ocrelizumab, B-cell depleted patients, SARS-CoV2 vaccination, Comirnaty®, T-cell mediated response, Lack of antibody response, Multiple sclerosis, COVID-19

## Abstract

**Background:**

Patients with multiple sclerosis receiving ocrelizumab-treatment are in desperate need of a protection against SARS-CoV-2 infection.

**Methods:**

In this study, Euroimmun semi-quantitative Anti-SARS-CoV-2 IgG for detection of humoral response and ELISpot assays for detection of SARS-CoV-2-specific T-cell-response were used in 10 ocrelizumab-treated patients with multiple sclerosis twice vaccinated with Comirnaty® mRNA vaccine. This data was compared with a control group of 20 age- and sex-matched healthy volunteers, who had all previously received a full SARS-CoV-2 mRNA vaccination with Comirnaty® or Spikevax®.

**Results:**

While all subjects in the control group had high humoral response to the vaccination, in B-cell-depleted individuals a significantly reduced antibody response to vaccination against SARS-CoV-2 was observed. SARS-CoV-2 specific T-cell-response, however, did not differ significantly between both cohorts.

**Conclusions:**

T-cell-mediated response to Comirnaty® vaccination is observable despite attenuated humoral response in B-cell-depleted patients. This might enable partial protection against COVID-19.

*Trial registration* Retrospectively registered.

## Background

The ongoing coronavirus disease 2019 (COVID-19) pandemic, caused by infection with severe acute respiratory syndrome coronavirus type 2 (SARS-CoV-2), is a major threat to global health and especially endangers individuals who suffer from chronic diseases such as multiple sclerosis (MS). While risk of hospitalization, intensive care and death due to COVID-19 in patients with multiple sclerosis (pwMS) is almost equal compared to healthy individuals [8, 17], patients undergoing disease modifying therapies and especially B-cell-depletion likely suffer from a more severe course of infection [9, 10, 16]. Sensitivity to more severe virus infections in B-cell-depleted patients is known to occur due to reduced humoral immunity, hampering effective virus clearance [12]. Hence, B-cell-depleted patients are in desperate need for protection against SARS-CoV-2 infection.

Another problematic aspect of B-cell-depletion is the compromised effectivity of vaccines, as demonstrated in the VELOCE study, where missing humoral response to various vaccines (such as tetanus and seasonal influenza vaccines) in ocrelizumab-treated patients was shown, leading to the recommendation to complete vaccination before initiation of ocrelizumab-therapy [4].

Therefore, the objective of this study was to investigate T-cell-response to SARS-CoV-2-vaccination in pwMS treated with ocrelizumab to gather information about humoral and cellular immunity in this fragile group of patients.

## Methods

### Volunteers

All patientsincluded in the study were recruited in the outpatient clinic for MS of the Alfried Krupp Hospital in Essen, Germany. All patients were treated with ocrelizumab (Ocrevus®) for MS; of these ten pwMS, eight had a relapsing remitting disease course and two were diagnosed with a primary progressive disease course. EDSS varied from 0 to 7. Ocrelizumab was assessed in a standard dose of 600 mg, but in terms of reducing social interaction of immunocompromised patients during COVID-19 pandemic, following cycles were not applied twice a year but only after B-cell-repopulation was detectable via FACS-analysis.

All patients included in this study were female. They had a mean age of 50 years (median 47, range 34–79). All received two vaccinations with Comirnaty® (BNT162b2, BioNTech/Pfizer). None reported symptoms of SARS-CoV-2 infection. The second vaccination was performed at a median of 34 days (range 25–46) prior to blood sampling.

The mean interval between last ocrelizumab cycle to first vaccination was 125 days (median 124, range 7–266) and to second vaccination 152 days (median 154, range 28–282), varying as many patients show long-term suppression of B-cells after ocrelizumab-application.

In most patients, B-cell-suppression was measured close to the time of vaccination. In three patients, B-cell count was 1% and in five patients, it was 0%. In two patients no FACS-analysis was performed, as ocrelizumab was applied shortly before vaccination and complete B-cell-suppression could be expected. In most patients (n = 6), FACS-analysis was performed between the two vaccinations, specifically 15 days (median 20, range 1–30) before the second vaccination. In one patient, FACS-analysis was done 8 days before first and 30 days before second vaccination. In another patient, FACS-analysis was sampled 28 days after second vaccination.

As a control group, we included 20 female healthy volunteers (mean age of 49 years, median 53, range 22–80), who had been vaccinated twice against SARS-CoV-2, without symptoms of SARS-CoV-2 infection before vaccination. Immunity of the vaccinated volunteers was analyzed at a median of 29 days (range 25–77) after the second vaccination. Seven of them were vaccinated with Comirnaty® (BioNTech/Pfizer) and 13 with Spikevax ® (mRNA-1273, Moderna Biotech).

As a further control for SARS-CoV-2 IgG antibodies, we tested 50 retention samples from our blood bank that were collected in November 2016.

The study was approved by the local ethics committee of the University Hospital Essen, Germany (20-9225-BO). All volunteers provided informed consent to participate in the study. This study has been performed in accordance with the ethical standards noted in the 1964 Declaration of Helsinki and its later amendments or comparable ethical standards. Data not provided in the article because of space limitations may be shared (anonymized) at the request of any qualified investigator for purposes of replicating procedures and results.

### ELISpot assay

To assess SARS-CoV-2-specific cellular immunity, we performed ELISpot assays, using peptide pools of the Spike (S) 1, the S1/S2, the membrane (M) and the nucleocapsid (NC) protein (Miltenyi Biotec, Bergisch Gladbach, Germany) and an S1 protein of SARS-CoV-2 (S Sino, Sino Biological, Wayne, PA, USA). Whereas responses to the spike are expected after SARS-CoV-2 vaccination and infection, responses to the nucleocapsid should only occur after infection. We used this peptide pool to exclude volunteers with prior infection. Responses to the membrane are not completely specific for SARS-CoV-2 infection, but could also occur as a cross-reaction after infection with human endemic coronaviruses. We tested duplicates of 250,000 peripheral blood mononuclear cells (PBMC) per cell culture and measured IFN-γ production after 19 h. SARS-CoV-2 specific spots were determined as stimulated minus non-stimulated (background) values (spots increment). We defined at least three spots above background together with threefold higher SARS-CoV-2 specific spots versus background as positive response. Thereby, the detection limit of our assay is in the range of 3/250,000 functionally active and SARS-CoV-2 specific cells. Details on the ELISpot assay and the cutoff definition have been published previously [14].

### Antibody ELISA

Antibodies were determined by a CE marked Anti-SARS-CoV-2 IgG semi-quantitative ELISA (Euroimmun, Lübeck, Germany), according to the manufacturers’ instructions. Results of S1 protein specific IgG antibodies are given as ratio (patient sample / control sample). An antibody ratio of > 1.1 was considered positive, of ≥ 0.8 to < 1.1 borderline and of < 0.8 negative.

### Statistical analysis

Statistical analysis was performed with GraphPad Prism 8.0.1 (San Diego, CA, USA). For the analysis of numerical variables, we used Spearman correlation and linear regression analysis. To assess the impact of categorical covariates we used Mann–Whitney test. Two-sided *p* values < 0.05 were considered significant.

## Results

**Fig. 1:**
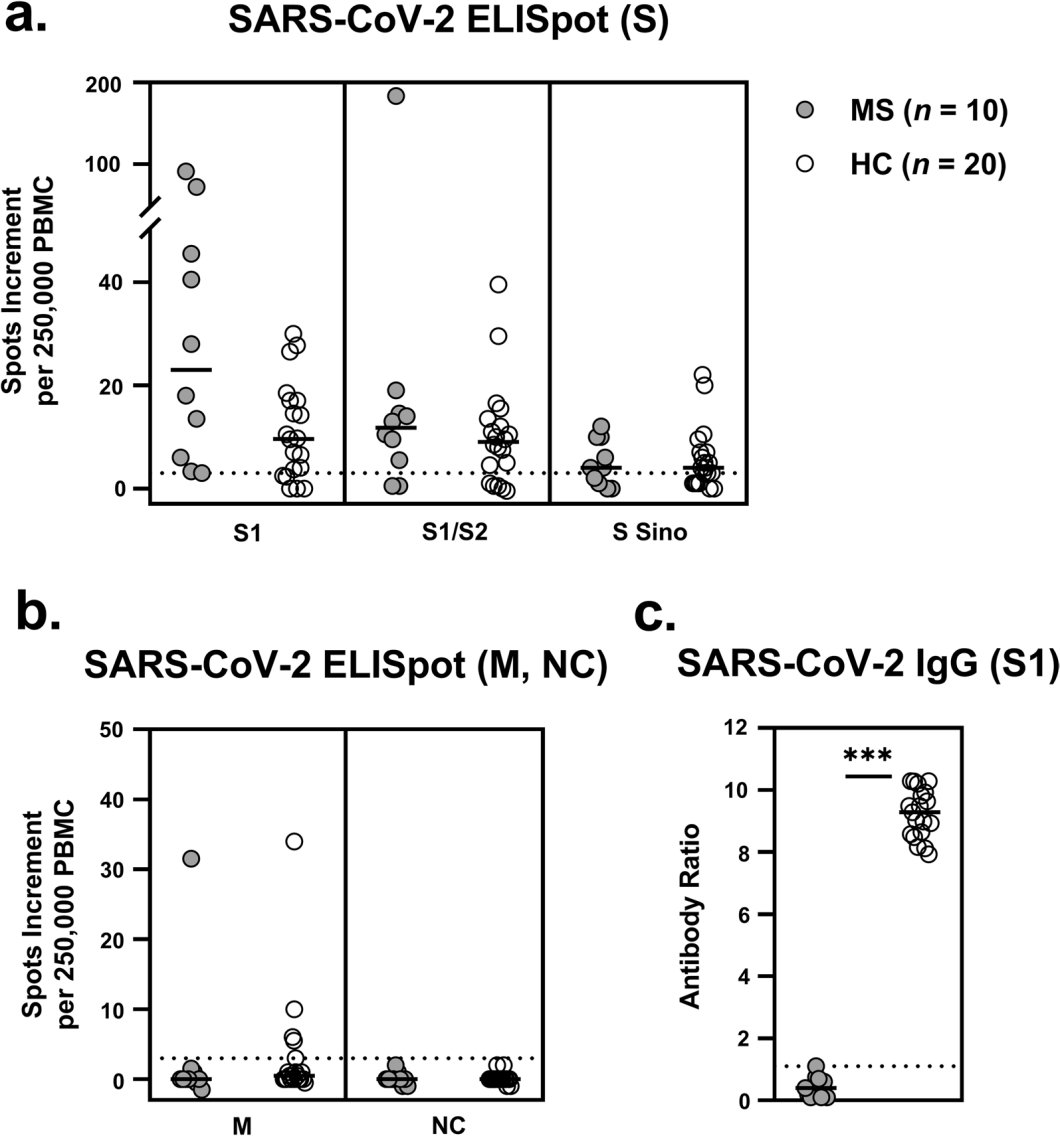
SARS-CoV-2 specific ELISpot and IgG responses in patients with multiple sclerosis (MS) and matched healthy controls (HC). **a** ELISpot responses to spike (S), **b** to membrane (M) and nucleocapsid (NC) and **c** IgG antibody responses against S1. Horizontal bold lines indicate median values, dashed lines the cutoff for positive responses (3 spots increment or antibody ratio of 1.1). S1: peptide mix of the SARS-CoV-2 spike (S) 1; S1/S2: peptide mix of the spike (S) 1 and S2; S Sino: S1 protein; M: peptide mix of the membrane; NC: peptide mix of the nucleocapsid. ***p < 0.0001 (Mann–Whitney test)

In all ten patients with multiple sclerosis who received the B-cell-depleting antibody ocrelizumab (Ocrevus®, anti-CD20 Ab) we observed—as expected—a significantly reduced antibody response to vaccination against SARS-CoV-2 (Fig. 1). The patients reached a median antibody ratio of 0.4 (range 0.1–1.1) and none of the responses could be classified as positive. In comparison, all 20 vaccinated, age- and sex-matched healthy controls showed detectable antibody responses, with a median S1-specific IgG ratio of 9.3 (range 7.9–10.3) (*p* < 0.0001). However, SARS-CoV-2 specific T cell responses did not differ significantly between both cohorts. In detail, ELISpot responses to the spike peptides S1 and S1/S2 were even slightly higher and to an S protein antigen (S Sino) were similar in patients with multiple sclerosis (S1: 23.0 vs. 9.6; S1/S2: 11.8 vs. 9.0; S Sino: 4.0 vs. 4.0, data represent median values of spots increment). Of note, also the 79 year-old female patient treated with Ocrelizumab developed strong T cell immunity. In the control experiments, none of the volunteers showed responses to the nucleocapsid, i.e., none had evidence of previous SARS-CoV-2 infection. Responses to a rather conserved coronavirus antigen, the membrane, were also similar in both cohorts.

**Fig. 2:**
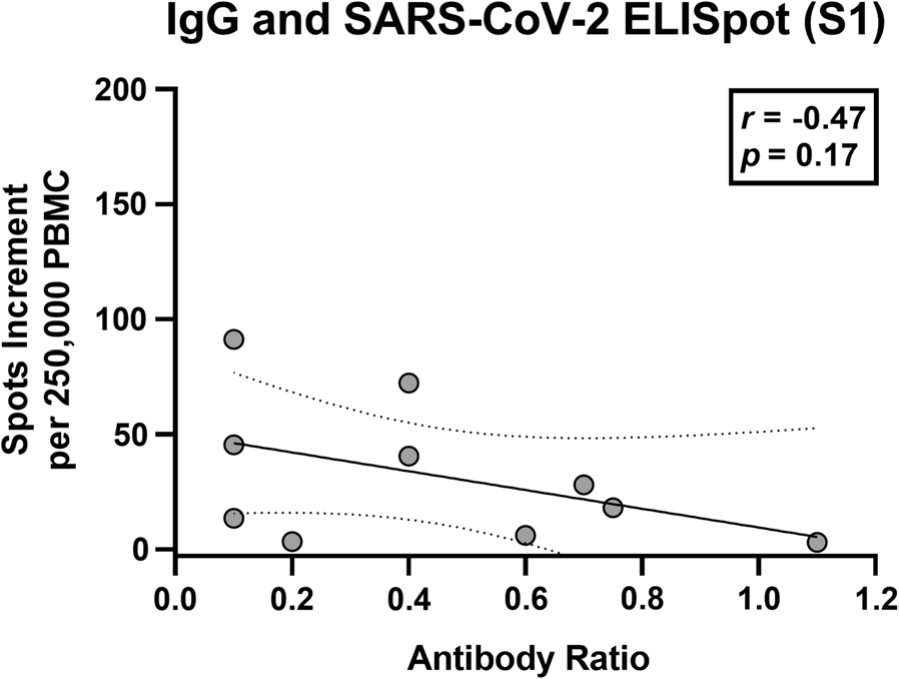
Spearman correlation analysis of SARS-CoV-2 specific IgG and Spike (S) 1 specific ELISpot responses in patients with multiple sclerosis (*n* = 10). The bold, continuous line indicates the regression line, the dashed lines the 95% confidence interval

Spearman analysis indicated that none of the SARS CoV-2 specific immune responses correlated significantly with age, the day after the second vaccination or the IgG ratio. There was even a trend for a negative correlation, when considering the S1 peptide mix (*r* = −0.47, *p* = 0.17) (Fig. 2). Of note, this peptide mix induced the strongest T-cell response. Interestingly, the difference between the patients and controls was most pronounced when using the S1 peptides as stimulus.

**Fig. 3:**
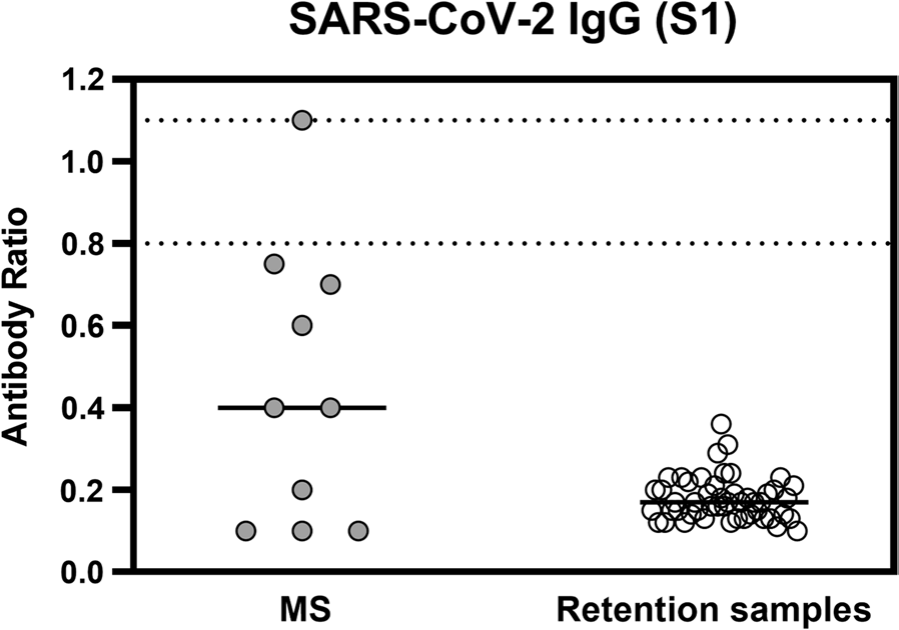
SARS-CoV-2 specific IgG responses in 10 patients with multiple sclerosis (MS) and 50 retention samples from 2016. Horizontal bold lines indicate median values, the dotted lines lower and upper limit of borderline responses (ratio of 0.8 and 1.1)

To find out if SARS-CoV-2 IgG antibodies were completely absent in the B-cell-depleted patients, we compared their antibody ratio with the ratio from 50 retention samples from our blood bank, that were collected in November 2016 (Fig. 3). Of note, the median antibody ratio in the patients was more than twofold higher (0.40 vs. 0.17, *p* = 0.08). However, this finding reached no statistical significance.

## Discussion

The current study indicates that B-cell-depletion in pwMS did not impair cellular immune responses after SARS-CoV-2 mRNA vaccination. Although all S1-specific IgG antibodies were below or at the cutoff for positive responses, they were higher than in retention samples from 2016.

As published recently, only 10 of 44 individuals (22.7%) of an Israeli cohort of pwMS receiving ocrelizumab-treatment developed humoral response to COVID-19 vaccine Comirnaty [1]. As individuals with B-cell repopulation had proper humoral response, postponement of the next therapy cycle in favour of an immune response to the COVID-19-vaccination has been recommended [3], eventually risking a higher occurrence of relapse in pwMS, especially since ocrelizumab is commonly prescribed in patients with highly active disease.

T-cell-mediated immunity might bypass such a dilemma because it is largely unaffected by CD19-depletion. As proof of principle, VZV-specific T-cell-response to VZV-vaccination was detected in patients receiving B-cell-depletion with rituximab in haematological diseases [13]. In 2017, Zhao et al. [18] already described the importance of T-cells for the recovery from a structurally related coronavirus, the Middle East respiratory syndrome (MERS) virus. In 2020, Braun et al. [7] speculated that T-cell immunity could also be protective against infection with SARS-CoV-2. Furthermore, SARS-CoV-2-specific T-cell-response was measured in antibody-seronegative family members of SARS-CoV-2-infected individuals [15]. There are now numerous publications confirming the importance of T-cell-responses for protection against SARS-CoV-2 [2, 6, 11].

It needs to be analyzed in a larger cohort of MS patients whether the strong T cell response just occurred randomly or whether it is a kind of compensation for the weak antibody response. We observed in approximately 15–20% of healthy controls only SARS-CoV-2 specific B or T cell immunity, despite PCR confirmed infection [14]. Thus, the immune response may be shifted more to an antibody or cellular response also in healthy individuals.

## Conclusion

In conclusion, this is the first published finding showing that, despite B-cell depletion due to Ocrelizumab treatment, pwMS who have been fully vaccinanated with Comirnaty® still show an extensive SARS-CoV 2 specific T-cell response. Similar data was recently shown in five patients treated with rituximab in rheumatic diseases [5]. Moreover, specific B-cell immunity was not completely absent and may provide some protection. However, this study has some limitations, specifically low number of patients and different intervals between Ocrelizumab and vaccination. Nevertheless, we suggest that vaccination under B-cell-depletion should be favored over delaying treatment cycles of ocrelizumab thus risking relapse after B-cell-repopulation.

## Data Availability

Data not provided in the article because of space limitations may be shared (anonymized) at the request of any qualified investigator for purposes of replicating procedures and results.

## Abbreviations

COVID-19: Coronavirus disease 2019
SARS-CoV-2: Acute respiratory syndrome coronavirus type 2
MS: Multiple sclerosis
pwMS: Patients with multiple sclerosis
EDSS: Expanded Disability Status Scale
PBMC: Peripheral blood mononuclear cells
FACS-analysis: Fluoresecence activated cell sorting
ELISpot: Enzyme Linked Immuno Spot Assay
ELISA: Enzyme-linked Immunosorbent Assay

## References

[1] Achiron A, Mandel M, Dreyer-Alster S, Harari G, Magalashvili D, Sonis P, Dolev M, Menascu S, Flechter S, Falb R, Gurevich M (2021). Humoral immune response to COVID-19 mRNA vaccine in patients with multiple sclerosis treated with high-efficacy disease-modifying therapies. Therapeutic Advances in Neurological Disorders.

[2] Altmann DM, Boyton RJ (2020). SARS-CoV-2 T cell immunity: Specificity, function, durability, and role in protection. Science Immunology.

[3] Baker D, Roberts CA, Pryce G, Kang AS, Marta M, Reyes S, Schmierer K, Giovannoni G, Amor S (2020). COVID-19 vaccine-readiness for anti-CD20-depleting therapy in autoimmune diseases. Clinical & Experimental Immunology.

[4] Bar-Or A, Calkwood JC, Chognot C, Evershed J, Fox EJ, Herman A, Manfrini M, McNamara J, Robertson DS, Stokmaier D, Wendt JK (2020). Effect of ocrelizumab on vaccine responses in patients with multiple sclerosis: The VELOCE study. Neurology.

[5] Bonelli MM, Mrak D, Perkmann T, Haslacher H, Aletaha D (2021). SARS-CoV-2 vaccination in rituximab-treated patients: Evidence for impaired humoral but inducible cellular immune response. Annals of the Rheumatic Diseases.

[6] Bonifacius A, Tischer-Zimmermann S, Dragon AC, Gussarow D, Vogel A, Krettek U, Gödecke N, Yilmaz M, Kraft AR, Hoeper MM, Pink I (2021). COVID-19 immune signatures reveal stable antiviral T cell function despite declining humoral responses. Immunity.

[7] Braun J, Loyal L, Frentsch M, Wendisch D, Georg P, Kurth F, Hippenstiel S, Dingeldey M, Kruse B, Fauchere F, Baysal E (2020). SARS-CoV-2-reactive T cells in healthy donors and patients with COVID-19. Nature.

[8] Fernandes PM, O’neill M, Kearns PK, Pizzo S, Watters C, Baird S, MacDougall NJ, Hunt DP (2020). Impact of the first COVID-19 pandemic wave on the Scottish Multiple Sclerosis Register population. Wellcome Open Research.

[9] Landtblom AM, Berntsson SG, Boström I, Iacobaeus E (2021). Multiple sclerosis and COVID-19: The Swedish experience. Acta Neurologica Scandinavica.

[10] Langer-Gould A, Smith JB, Li BH (2021). Multiple sclerosis, rituximab, and COVID-19. Annals of Clinical and Translational Neurology.

[11] Le Bert N, Tan AT, Kunasegaran K, Tham CY, Hafezi M, Chia A, Chng MH, Lin M, Tan N, Linster M, Chia WN (2020). SARS-CoV-2-specific T cell immunity in cases of COVID-19 and SARS, and uninfected controls. Nature.

[12] Misumi I, Whitmire JK (2014). B cell depletion curtails CD4+ T cell memory and reduces protection against disseminating virus infection. Journal of Immunology.

[13] Parrino J, McNeil SA, Lawrence SJ, Kimby E, Pagnoni MF, Stek JE, Zhao Y, Chan IS, Kaplan SS (2017). Safety and immunogenicity of inactivated varicella-zoster virus vaccine in adults with hematologic malignancies receiving treatment with anti-CD20 monoclonal antibodies. Vaccine.

[14] Schwarzkopf S, Krawczyk A, Knop D, Klump H, Heinold A, Heinemann FM, Thümmler L, Temme C, Breyer M, Witzke O, Dittmer U (2021). Cellular IMMUNITY in COVID-19 convalescents with PCR-confirmed infection but with undetectable SARS-CoV-2-specific IgG. Emerging Infectious Diseases.

[15] Sekine T, Perez-Potti A, Rivera-Ballesteros O, Strålin K, Gorin JB, Olsson A, Llewellyn-Lacey S, Kamal H, Bogdanovic G, Muschiol S, Wullimann DJ (2020). Robust T cell immunity in convalescent individuals with asymptomatic or mild COVID-19. Cell.

[16] Sormani MP, De Rossi N, Schiavetti I, Carmisciano L, Cordioli C, Moiola L, Radaelli M, Immovilli P, Capobianco M, Trojano M, Zaratin P (2021). Disease-modifying therapies and coronavirus disease 2019 severity in multiple sclerosis. Annals of Neurology.

[17] Zabalza A, Cárdenas-Robledo S, Tagliani P, Arrambide G, Otero-Romero S, Carbonell-Mirabent P, Rodriguez-Barranco M, Rodríguez-Acevedo B, Restrepo Vera JL, Resina-Salles M, Midaglia L (2020). COVID-19 in multiple sclerosis patients: Susceptibility, severity risk factors and serological response. European Journal of Neurology.

[18] Zhao J, Alshukairi AN, Baharoon SA, Ahmed WA, Bokhari AA, Nehdi AM, Layqah LA, Alghamdi MG, Al Gethamy MM, Dada AM, Khalid I (2017). Recovery from the Middle East respiratory syndrome is associated with antibody and T-cell responses. Science Immunology.

